# Predictive value of adipokines for the severity of acute pancreatitis: a meta-analysis

**DOI:** 10.1186/s12876-024-03126-w

**Published:** 2024-01-13

**Authors:** Xuehua Yu, Ning Zhang, Jing Wu, Yunhong Zhao, Chengjiang Liu, Gaifang Liu

**Affiliations:** 1 Hebei North University, Zhangjiakou, 075132 China; 2 Department of Gastroenterology, Hebei General Hospital, No.348, Heping West Road, Shijiazhuang, Hebei Province 050057 China; 3 Hebei Medical University, Shijiazhuang, 050011 China; 4 Department of Gastroenterology, Anhui Medical University, He Fei, 230601 China

**Keywords:** Adipokines, Acute pancreatitis, Resistin, Meta-analysis

## Abstract

**Background:**

Severe acute pancreatitis (SAP) is a dangerous condition with a high mortality rate. Many studies have found an association between adipokines and the development of SAP, but the results are controversial. Therefore, we performed a meta-analysis of the association of inflammatory adipokines with SAP.

**Methods:**

We screened PubMed, EMBASE, Web of Science and Cochrane Library for articles on adipokines and SAP published before July 20, 2023. The quality of the literature was assessed using QUADAS criteria. Standardized mean differences (SMD) with 95% confidence intervals (CI) were calculated to assess the combined effect. Subgroup analysis, sensitivity analysis and publication bias tests were also performed on the information obtained.

**Result:**

Fifteen eligible studies included 1332 patients with acute pancreatitis (AP). Pooled analysis showed that patients with SAP had significantly higher serum levels of resistin (SMD = 0.78, 95% CI:0.37 to 1.19, z = 3.75, *P* = 0.000). The difference in leptin and adiponectin levels between SAP and mild acute pancreatitis (MAP) patients were not significant (SMD = 0.30, 95% CI: -0.08 to 0.68, z = 1.53, *P* = 0.127 and SMD = 0.11, 95% CI: -0.17 to 0.40, z = 0.80, *P* = 0.425, respectively). In patients with SAP, visfatin levels were not significantly different from that in patients with MAP (SMD = 1.20, 95% CI: -0.48 to 2.88, z = 1.40, *P* = 0.162).

**Conclusion:**

Elevated levels of resistin are associated with the development of SAP. Resistin may serve as biomarker for SAP and has promise as therapeutic target.

**Supplementary Information:**

The online version contains supplementary material available at 10.1186/s12876-024-03126-w.

## Introduction

Acute pancreatitis (AP) is a common gastroenterological condition, with approximately 80% of patients developing mild to moderately severe disease (no organ failure > 48 h) and the rest progressing into severe acute pancreatitis (SAP) [1]. The death rate of SAP is as high as 20%, therefore, early assessment of severity in AP is crucial. Despite the large number of studies exploring early prediction of AP severity [2, 3], no ideal multifactorial scoring system and/or biochemical markers have been identified for early assessment of AP severity [4]. Therefore, early identification of the development of severe AP remains a great challenge.

In clinical studies, the components of metabolic syndrome have been found to be associated with the occurrence and deterioration of AP [5, 6]. In particular, obesity is an independent risk factor for the AP morbidity and mortality [7–10]. Depending on its location, adipose tissue can be divided into subcutaneous adipose tissue (SAT) and visceral adipose tissue (VAT). SAT, accounting for approximately 80% of all adipose tissue, acts as a reservoir for excess lipids. However, once the storage capacity is exceeded, which can only accommodate a limited number of adipocytes with limited expandability, fat begins to accumulate in areas outside the SAT, such as the liver, heart, skeletal muscles, and other sites [11, 12]. Numerous studies have shown that VAT, associated with the occurrence and development of AP [13–15], is a key site of inflammation and responsible for driving the systemic inflammatory response and exacerbating AP [16, 17], thus serving as an important prognostic indicator of AP severity. Being highly metabolically active, VAT can continuously release adipokines such as resistin, leptin, adiponectin, and visfatin into the portal circulation [18], which may involve in the development and progression of AP by modulating oxidative stress and inflammatory responses and influencing the severity of AP. Furthermore, resistin, leptin, adiponectin, and visfatin are well-known biomarkers for Nonalcoholic Fatty Liver Disease (NAFLD), which is a strong risk factor for AP and SAP.

Resistin has been found to increase the production of pro-inflammatory cytokines such as TNF-α, IL-1β, and IL-6 in mononuclear cells and macrophages [19, 20]. Additionally, it stimulates the production of cell adhesion molecules, including vascular cellular adhesion molecule-1 (VCAM-1), intercellular adhesion molecule-1 (ICAM-1), and monocyte chemoattractant protein (MCP)-1, as well as chemokine (C-C motif) ligand 2 (CCL 2), which contribute to chemotaxis and leukocyte recruitment to sites of inflammation [21, 22]. Leptin, which is mainly secreted by adipocytes, is a potent chemoattractant for immune cells, causing monocytes and macrophages to accumulate towards adipose tissue, and promoting increased expression of the inflammatory cytokines IL-6 and tumor necrosis factor (TNF) as well as toll-like receptor 4 (TLR4) [23]. At the same time, leptin is required for T-cell development and promotes the production of pro-inflammatory cytokines in CD4[+] T cells [24–26]. Adiponectin, a hormone mainly produced by white adipose tissue, can inhibit M1 macrophage activation [27, 28], exert anti-inflammatory effects by regulating JmJC family histone demethylase 3, which contributes to M2 polarization [29], and inhibit macrophage infiltration [30]. In animal studies, adiponectin-deficient mice exhibited more severe AP than wild-type mice, and adiponectin overexpression reduced the severity of AP [31]. Administration of exogenous recombinant adiponectin to AP mice significantly reduced NF-kB activity, cytokine levels, and tissue damage [32]. Visfatin has nicotinamide phosphoribose transferase (Nampt) activity, the rate-limiting enzyme of the nicotinamide adenine dinucleotide (NAD) salvage synthesis pathway, and macrophages rely on the NAD salvage pathway to meet their energy requirements and maintain their pro-inflammatory phenotype. Visfatin also promotes the release of pro-inflammatory cytokines IL-1β, IL-6, and TNF-α from peripheral monocytes [33–35].

Despite many studies that have explored the relationship between adipokines and SAP, the findings have been inconsistent. Furthermore, even though a meta-analysis of the relationship between adipokines and SAP has recently been published [36], it only examined the statistical correlation between resistin and SAP, without addressing the correlation between other adipokines and SAP. Therefore, we performed this meta-analysis, involving such adipokines as resistin, leptin, adiponectin, and visfatin, to explore the correlation between adipokines and SAP.

## Method

This study was performed in accordance with the Preferred Reporting Items for Systematic Review and Meta-Analysis (PRISMA) guidelines.

### Search strategy

We conducted a systematic literature search on Embase, Cochrane library, PubMed and Web of Science, using the following keywords: (“adipokines”, “resistin”, “leptin”, “visfatin” or “adiponectin”) AND (“acute pancreatitis”) and MeSH/Emtree terms as well (Table S1). The deadline for the search was July 20, 2023. In addition, we checked the references of the screened literature to identify any additional relevant studies.

### Study selection

Inclusion criteria: (1) study subjects with a confirmed diagnosis of AP were included; (2) the severity of the AP was assessed; (3) the concentration of resistin, leptin, endolipin or lipocalin in peripheral blood was measured; (4) complete data calculation metrics were available: including the mean of the concentrations of resistin, leptin, visfatin or adiponectin with corresponding standard deviations (SD) or 95% confidence intervals (CI); (5) studies republished after additional data in the literature on the same topic, using the most recent study data.

Exclusion criteria: (1) duplicate articles; (2) reviews, meta-analyses, editorials, and letters; (3) animal studies or in vitro experiments; (4) articles whose data were unavailable; (5) studies that were subgroup analyses of included multicenter studies.

Both the study selection and exclusion procedures described above were conducted by two independent investigators (Xuehua Yu and Ning Zhang). Once disagreements occur, a third independent reviewer (Jing Wu) was invited to make the final decision.

### Data extraction and quality assessment

Data were extracted and cross-checked independently by two authors (Xuehua Yu and Ning Zhang) using a pre-developed data extraction form, and in case of disagreement, they were referred to a third investigator (Yunhong Zhao) for verification. Extractions included: first author, year of publication, country, types of adipokines, the time of the blood test, assay method, AP diagnostic criteria, sample size, sample characteristics, etiology, adipokine concentration (mean, SD), and fund.

To evaluate the risk of bias and quality of all included studies, we used the Quality Assessment of Diagnostic Accuracy Studies tool (QUADAS) [26], which was adapted to the studies included in this meta-analysis. All assessments were performed by two independent investigators (Xuehua Yu and Ning Zhang), and any disputes were resolved through consultation or discussion with a third party (Chengjiang Liu).

### Statistical analysis

Continuous outcomes measured on the same scale were expressed as a mean value and standard deviation and were analyzed by using standardized mean difference (SMD). Statistical analyses of heterogeneity were conducted using the chi-squared Q test and the I-square (*I*^*2*^) statistic. *P* < 0.10 and *I*^*2*^ > 50% were considered statistically significant heterogeneity thresholds. Calculation of the pooled SMD was performed using a random effects model. Moreover, subgroup and sensitivity analyses were used to further explore the sources of heterogeneity. All *P*-values were 2-tailed, and *P* < 0.05 (except for tests of heterogeneity) was considered statistically significant. Publication bias was assessed by Egger’s test and Begg’s test.

## Results

### Literature search and research characteristics

**Fig. 1:**
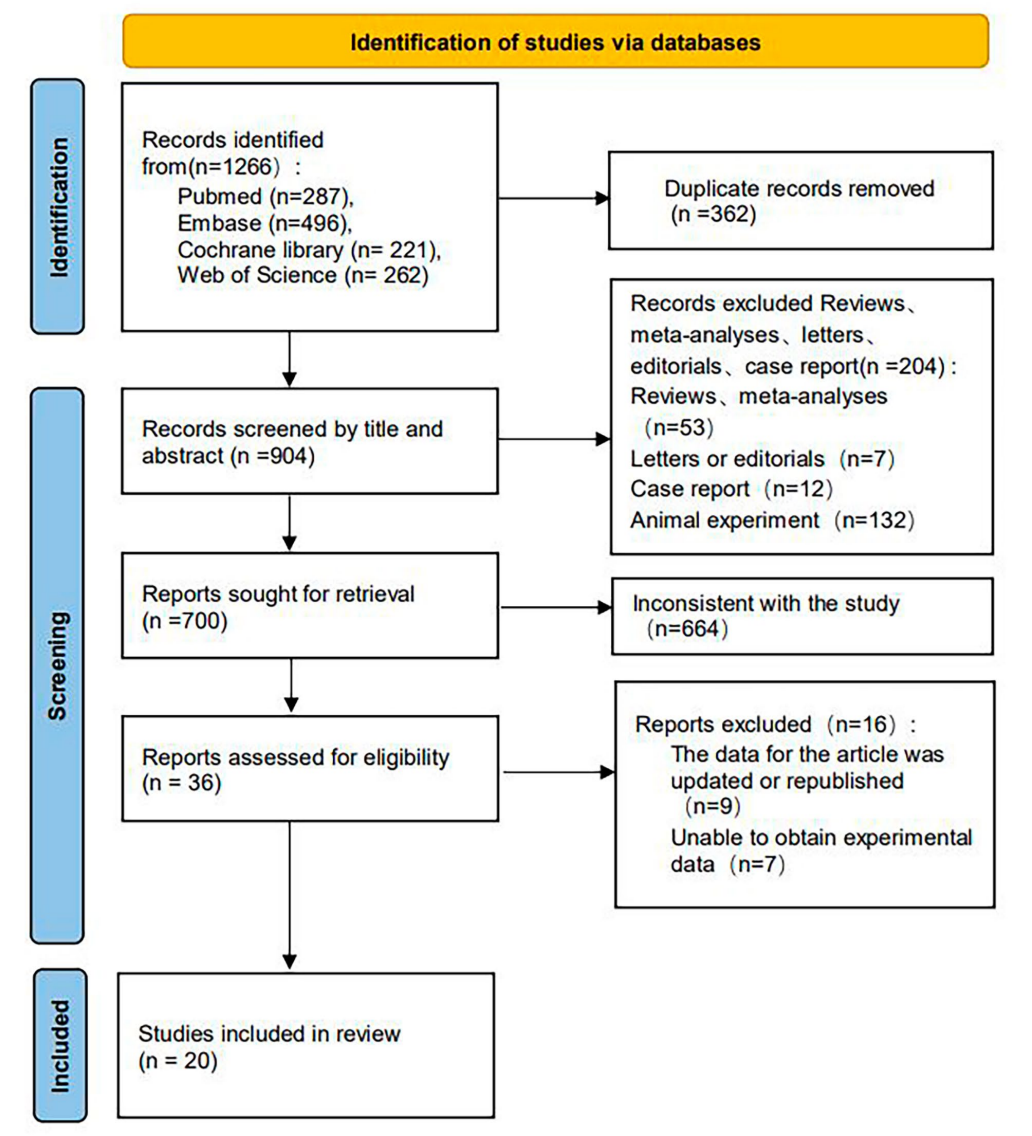
The PRISMA flow chart of literature screening

According to a predefined search strategy, we searched PubMed, EMBASE, Web of Science, and Cochrane Library, generating 1266 articles. By strictly following the inclusion and exclusion criteria, 20 articles were finally included, and the specific screening process is shown in Fig. 1.

The main characteristics of the included studies are summarized in Table 1, Table S2, S3 and S4. A total of 1332 AP patients were evaluated in studies conducted in countries (4 in Turkey, 3 in the United States, 3 in China, 2 in Czech Republic, 2 in Germany, 1 in India, 1 in México, 1 in Poland, 1 in Finland, 1 in Saudi Arabia, and 1 in Lithuania). Among the 20 studies, 10 evaluated the predictive effect of resistin on SAP, 8 focused on the predictive effect of leptin on SAP, 7 evaluated the predictive effect of adiponectin on SAP, and 3 investigated the predictive effect of visfatin on SAP. The detailed statistics of each adipocytokine are shown in Table 2. The quality assessment of all included studies that applied the QUADAS risk of bias assessment tool is shown in Table S5.

**Table 1:** Characteristics of 20 studies included in the meta-analysis (1)

Author, year	Country	Assay Method				Sample size,n		Collecting time	Etiology
		Resistin	Leptin	Adiponectin	Visfatin	SAP	MAP		
Kisaoglu,2014 [67]	Turkey	ELISA	-	-	-	17	17	on the 1st day	n/r
Schäffler A, 2010 [68]	Germany	ELISA	ELISA	ELISA	-	41	9	at admission	gallstones, alcohol, metabolic, ERCP, toxic,
Kibar YI, 2016 [69]	Turkey	ELISA	-	-	-	22	37	at admission	biliary/nonbiliary
Singh AK, 2021 [70]	India	ELISA	-	-	-	53	77	at admission	alcohol, gallstone, others
Karpavicius A, 2016 [71]	Lithuania	ELISA	ELISA	ELISA	ELISA	20	82	at admission	biliary stones, alcohol, others
Al-Maramhy, 2014 [72]	Saudi Arabia	ELISA	-	-	-	22	80	at admission	gallstone
Yu P, 2016 [73]	China	Luminex xMAP	ELISA	Luminex xMAP	-	24	66	at admission	biliary, alcoholic, hypertriglyceridaemia, others
Muddana V, 2010 [74]	America	Luminex assay	-	-	-	19	27	in early time	n/r
Novotny D, 2015 [75]	Czech Republic	-	-	ELISA	-	14	70	at admission	alcohol, biliary, CHP (chronic pancreatitis) exacerbation, idiopathic, others
Sharma A, 2009 [76]	America	-	-	IF	-	10	26	days 1 to 3	n/r
Tukiainen E, 2006 [77]	Finland	-	IA	IA	-	12	12	at admission	alcohol, biliary, idiopathic
Türkoğlu A, 2014 [78]	Turkey	-	ELISA	-	-	30	62	within 24 h of admission	biliary, alcoholic, hypertriglyceridemia, idiopathic, ERCP
Panek J, 2014 [79]	Poland	-	RIA	-	-	11	9	1st	biliary
Duarte-Rojo A, 2006 [80]	México	-	ELISA	-	-	14	38	at admission	biliary, hypertriglyceridemia, alcoholic, ERCP, others
Schäffler A, 2011 [81]	Germany	-	-	-	ELISA	41	9	at admission	gallstones, alcohol, metabolic, ERCP, toxic,
Ülger BV, 2014 [82]	Turkey	-	ELISA	-	-	8	32	at admission	gallstones
Deng LH, 2017 [83]	China	Human Obesity Premixed Kit	-	-	-	20	50	within 24 h of admission	biliary, hypertriglyceridemia, alcoholic, others
Langmead C, 2021 [84]	America	custom human duplex kits	-	-	-	37	96	at days 2, 3, and 4	biliary, alcoholic, idiopathic, other
Malina P, 2014 [85]	Czech Republic	-	-	ELISA	-	10	43	at admission	biliary, alcoholic, exacerbation of chronic pancreatitis, idiopathic
Guo F, 2021 [86]	China	-	-	-	ELISA	30	35	at admission	biliary, alcoholic, other

n/r, not reported; ELISA, Enzyme Linked Immunosorbent Assay; IA, immunoassays; IF, immunofluorescence

**Table 2:** Circulating levels of resistin, leptin, adiponectin and visfatin in SAP and MAP patients

Author, year	SAP				MAP			
	Mean	SD	unit	N	Mean	SD	unit	N
**Circulating resistin levels**								
Kisaoglu, 2014 [67]	26.48	12.03	ng/dl	17	23.50	12.30	ng/dl	17
Schäffler A, 2010 [68]	74.1	94.9	ng/ml	41	35.9	54.6	ng/ml	9
Kibar YI, 2016 [69]	28.9	5.22	ng/ml	22	18.3	6.95	ng/ml	37
Singh AK, 2021 [70]	1.24	1.72	ng/ml	53	1.39	2.45	ng/ml	77
Karpavicius A, 2016 [71]	20.2	31.75	ng/ml	20	10.7	8.65	ng/ml	82
Al-Maramhy, 2014 [72]	17.5	0.96	ng/ml	22	16.82	1.10	ng/ml	80
Yu P, 2016 [73]	230.94	215.79	ng/ml	24	107.95	85.76	ng/ml	66
Muddana V, 2010 [74]	51,316	59023.7	pg/ml	19	7504	4199.26	pg/ml	27
Deng LH, 2017 [83]	53264.28	125153.22	n/r	20	11686.23	59253.78	n/r	50
Langmead C, 2021 [84]	12,104	10,359	n/r	37	2175	2089	n/r	96
**Circulating leptin levels**								
Schäffler A, 2010 [68]	20.9	30.7	ng/ml	41	17.5	13.9	ng/ml	9
Karpavicius A, 2016 [71]	4.17	8.14	ng/ml	20	7.21	11.83	ng/ml	82
Yu P, 2016 [73]	7.07	6.61	ng/ml	24	5.01	6.48	ng/ml	66
Tukiainen E, 2006 [77]	6.1	52.8	ng/ml	12	9.0	25.2	ng/ml	12
Türkoğlu A, 2014 [78]	9.32	5.80	ng/ml	30	4.69	3.46	ng/ml	62
Panek J, 2014 [79]	24.7	20.0	ng/ml	11	6.8	6.7	ng/ml	9
Duarte-Rojo A, 2006 [80]	10.3	44.8	ng/ml	14	7.7	158.9	ng/ml	38
Ülger BV, 2014 [82]	7.63	6.27	ng/ml	8	6.93	4.35	ng/ml	32
**Circulating adiponectin levels**								
Schäffler A, 2010 [68]	12.2	20.8	µg/ml	41	9.9	8.1	µg/ml	9
Sharma A, 2009 [76]	3.74	5.99	µg/L	10	6.58	10.41	µg/L	26
Karpavicius A, 2016 [71]	7.91	10.07	ng/ml	20	11.10	9.58	ng/ml	82
Yu P, 2016 [73]	13.65	8.08	ng/ml	24	10.17	11.34	ng/ml	66
Novotny D, 2015 [75]	8.3	2.6	mg/L	14	7.4	3.0	mg/L	70
Tukiainen E, 2006 [77]	5642	13,481	ng/ml	12	6314	16,563	ng/ml	12
Malina P, 2014 [85]	8.45	1.56	mg/L	10	6.4	3.0	mg/L	43
**Circulating visfatin levels**								
Schäffler A, 2011 [81]	6.8	9.6	ng/ml	41	3.3	1.5	ng/ml	9
Karpavicius A, 2016 [71]	5.42	4.74	ng/ml	20	4.15	5.45	ng/ml	82
Guo F, 2021 [86]	10.75	2.92	ng/ml	30	3.70	1.73	ng/ml	35

SAP, Severe acute pancreatitis; MAP, Mild acute pancreatitis; SD, standard deviation; N, number; n/r, not reported

### Relationship between adipokines and SAP

**Fig. 2:**
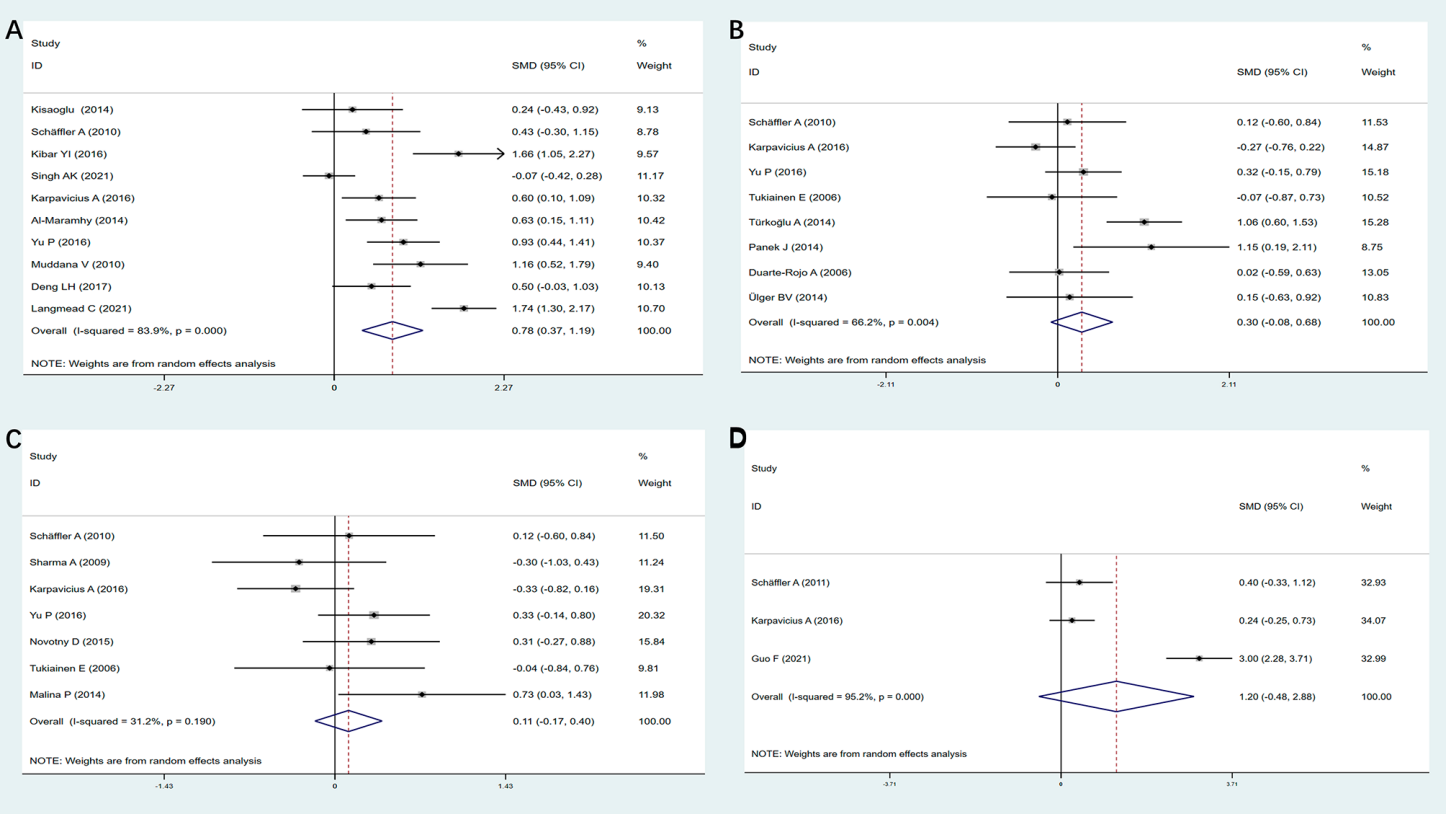
Forest plots of SMD with 95% CI of peripheral blood levels of resistin (**A**), leptin (**B**), adiponectin (**C**) and visfatin (**D**) levels between SAP patients and MAP patients

A total of 7 of 10 studies showed significantly increased levels of resistin in patients with SAP relative to patients with mild acute pancreatitis (MAP). A total of 275 SAP patients and 541 MAP patients were included in the summary analysis, as shown in Fig. 2A. The pooled analysis showed significantly higher resistin levels in SAP patients as compared to MAP patients (SMD = 0.78, 95% CI:0.37 to 1.19, z = 3.75, *P* = 0.000). However, statistically significant heterogeneity was observed in these studies (*P* = 0.000, *I*^*2*^ = 83.9%).

For leptin, 3 out of 8 studies saw significantly higher levels in patients with SAP. A total of 160 SAP patients and 310 MAP patients were analyzed. Leptin levels were not significantly higher in SAP patients than in MAP patients (SMD = 0.30, 95% CI: -0.08 to 0.68, z = 1.53, *P* = 0.127) (Fig. 2B). Again, significant heterogeneity was observed in the study (*P* = 0.004, *I*^*2*^ = 66.2%).

A total of 1 out of 7 studies showed significantly lower adiponectin levels in patients with SAP as compared to those with MAP. Pooled analysis showed no significant difference in adiponectin levels between 131 SAP patients and 308 MAP patients (SMD = 0.11, 95% CI: -0.17 to 0.40, z = 0.80, *P* = 0.425) (Fig. 2C). Significant heterogeneity was found in these 10 studies (*P* = 0.190, *I*^*2*^ = 31.2%).

Only 3 studies have examined blood visfatin levels in SAP patients and MAP patients. A total of 91 patients with SAP and 126 patients with MAP were analyzed. Visfatin levels were not significantly higher in patients with SAP than in those with MAP (SMD = 1.20, 95% CI: -0.48 to 2.88, z = 1.40, *P* = 0.162) (Fig. 2D). Again, significant heterogeneity was observed in the study (*P* = 0.000, *I*^*2*^ = 95.2%).

### Subgroup analysis

According to year of publication, sample size, mean age of patients, and definition of SAP group and MAP group (Table S4), subgroup analysis was performed to explore the impact of these three factors on outcomes as well as to identify potential sources of resistin and leptin heterogeneity.

As shown in Fig S1A, pooled results from the literature published before 2014 and in 2014 and after showed that resistin was predictive of SAP. Pooled results from studies in which the mean age of patients with AP was < 50 years versus age ≥ 50 years also indicated that resistin was a predictor of SAP (Fig S1B). Studies with a sample size of < 100 patients showed significantly higher resistin levels in SAP patients than in MAP patients (SMD = 0.83, 95% CI: 0.42 to 1.24, z = 3.98, *P* = 0.000, *I*^*2*^ = 64.1%, Fig S1C), but studies having a sample size of ≥ 100 patients showed no statistically significant difference in resistin levels between the two groups (SMD = 0.72, 95% CI: -0.08 to 1.52, z = 1.77, *P* = 0.076, *I*^*2*^ = 92.6%, Fig S1C). In addition, SAP was defined as persistent organ failure (> 48 h) in 6 studies that tested resistin levels, showing a significant difference between in the MAP group and the SAP group (SMD = 0.80, 95% CI: 0.23 to 1.37, z = 2.73, *P* = 0.006, *I*^*2*^ = 88.7%, Fig S1D).

Regarding leptin, as shown in Fig S2, different publication years, ages, and definition of SAP and MAP showed no statistically significant difference in leptin levels between the two groups. However, the pooled results of the 7 studies with sample sizes < 100 showed that leptin levels were higher in the SAP group than in the MAP group, and the difference was statistically significant (SMD = 0.40, 95% CI: 0.02 to 0.77, z = 2.07, *P* = 0.038, *I*^*2*^ = 57.2%, Fig S2C).

There were no statistically significant differences in lipocalin levels between the two groups for different publication years, sample sizes, ages, and definitions of SAP and MAP, as shown in Figure S3.

### Sensitivity analysis

**Fig. 3:**
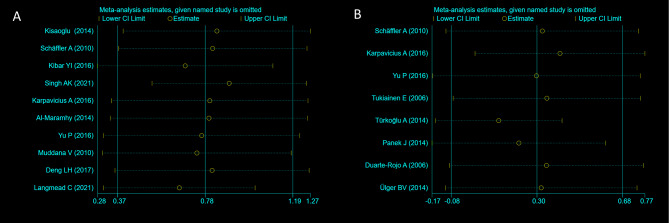
The pooled SMD and 95%CI of eligible studies of resistin (**A**) and leptin (**B**) through sensitivity analysis

Sensitivity analysis was performed whereby each study was excluded in turn to assess the stability of the results and the impact of each study on the pooled SMD was also determined (Fig. 3). It can be seen from Fig. 3A that the studies by Kibar YI et al., Singh AK et al. and Langmead C et al. had the greatest influence on the results regarding resistin. Although these 3 studies were removed, SAP patients showed significantly higher resistance levels than MAP patients (SMD = 0.66, 95% CI: 0.45 to 0.87, z = 6.21, *P* = 0.000, *I*^*2*^ = 0.0%, Fig S4). As shown in Fig. 3B, the results of the study by Türkoğlu A et al. had the greatest impact on the results regarding leptin, and removal of this study still showed no significant increase in leptin levels in SAP patients compared to MAP patients (SMD = 0.13, 95% CI: -0.15 to 0.41, z = 0.88, *P* = 0.379, *I*^*2*^ = 23.9%, Fig S5).

### Publication bias

For resistin, leptin and lipocalin, symmetry was observed in Begg’s funnel plot (Fig S6), with Egger’s test results *(P* = 0.444, *P* = 0.869, *P* = 0.920, respectively, Fig S7), suggesting no publication bias.

## Discussion

The results of this meta-analysis showed that increased resistin levels were associated with SAP, whereas leptin and adiponectin levels were not linked to SAP. Only three of the studies included visfatin, not enough to draw any conclusions.

Resistin is a small protein rich in cysteine, with a molecular weight of either 11 or 12.5 kDa. It was first identified in mice in 2001 as a signal molecule produced by adipocytes, and named resistin because it was thought to be involved in the development of insulin resistance [37]. Resistin belongs to the resistin-like molecule (RELM) family, which includes RELM-α, RELM-β, and RELM-γ [38]. Unlike mice, where resistin is produced by adipocytes, humans mainly express resistin in monocytes and macrophages [39]. Despite only sharing 59% of the same amino acids [40], resistin functions similarly in both humans and rodents, even though they are produced from different sources. Resistin has been identified as a molecule that promotes inflammation and regulates various chronic inflammatory, metabolic, and infectious diseases in humans [41–44]. It modulates many cellular responses in the host, such as recruiting and activating immune cells, promoting the release of pro-inflammatory cytokines, enhancing interferon (IFN) expression, and promoting the formation of neutrophil extracellular trap networks (NETs) [45–47].

The role of resistin in regulating inflammatory pathways has been demonstrated in the context of AP. Resistin increases the levels of calcium in pancreatic follicular cells, as well as the activity of NADPH oxidase, leading to an increase in the production of reactive oxygen species (ROS) within the cells. Additionally, resistin activates the NF-κB pathway, resulting in the expression of pro-inflammatory cytokines such as TNF-α and IL-6 [48, 49]. Jiang et al. demonstrated in a laboratory model of AP induced by cerulein that resistin increases the production of pro-inflammatory cytokines TNF-α and IL-6 via an NF-κB-dependent pathway. However, the increased mRNA expression levels of TNF α and IL 6 induced by resistin can be significantly reduced by using an NF-κB inhibitor [50]. Furthermore, Wang et al. discovered that the severity of SAP lung injury was positively associated with RELMα levels. Moreover, overexpression of RELMα worsened the release of inflammatory cytokines such as interleukin (IL)-1β, IL-6, IL-8, tumor necrosis factor-α, and serum C-reactive protein. This led to an increase in the expression of inflammatory mediators such as phosphorylated (p)-AKT, p-P65, p-P38 mitogen activated protein kinase, p-extracellular regulated kinase, and intracellular adhesion molecule-1, ultimately resulting in lung injury. On the other hand, knocking down RELMα had the opposite effect. It improved the expression of proliferative cellular nuclear antigen, Bcl-2, zonal occludin-1, and Claudin-1 in lung tissue of SAP rats [51]. Furthermore, numerous studies have confirmed the correlation between resistin and the severity of AP. This suggests that resistin may serve as a valuable marker and potential therapeutic target for SAP [52].

Leptin is mainly secreted by fat cells and plays a crucial role in the immune response as an immune modulator [53, 54]. Monocytes treated with leptin increase the production of type 1 cytokines, including IL-1β, IL-6, TNF, and resistin [55, 56]. Adiponectin can inhibit the ROS/NF-κB/NLRP3 inflammatory pathway [57], activate the anti-inflammatory cytokine interleukin-10 (IL-10), and reduce pro-inflammatory cytokines such as interferon-gamma (IFN-γ), IL-6 and TNF-α in human macrophages [58]. The results of this meta-analysis showed that leptin and adiponectin levels were not linked to SAP. However, it is still unclear whether leptin and adiponectin have different effects at different stages of inflammation, or whether an imbalance among leptin, adiponectin and other adipokines may inhibit their regulation of the immune response, or whether there are other possible mechanisms, which need to be confirmed by more studies. Although most studies show that visfatin appears to have pro-inflammatory effects [33–35, 59–62], there are some studies that show the opposite [29, 30, 63]. In response to this seemingly contradictory result, the study by Sayers et al. may give us some insight. They found a possible bimodal effect of extracellular Nampt (eNampt) monomer on the stimulation of insulin secretion by β-cells [64]. Whether this bimodal effect is equally reflected in the stimulatory effect of endolipin on inflammatory factors and the modulation of the inflammatory response, and whether it is this bimodal effect that leads to the unstable prediction of SAP by visfatin, remain to be further explored.

Heterogeneity was observed in our pooled analysis. The resistin results were greatly influenced by two studies, while the leptin results were mainly affected by one study. Several factors such as regions, research samples, and detection reagents can affect the outcomes. Small sample sizes can also lead to accidental findings, making heterogeneity between studies inevitable. However, the stability of the results was confirmed even after removing the heterogeneous studies. Furthermore, sample size and mean age of the patients may be associated with resistin heterogeneity. It has been shown that the adverse effects of obesity appear to be reduced in older populations [65]. Khatua et al. suggested that the different visceral triglyceride saturation status could have varying effects on AP severity, explaining the obesity paradox [66]. Based on the results of the subgroup analysis in this meta-analysis, it appears that the mean age of patients has an effect on adiposity factors and resultantly affects AP severity, which may provide a new thought for the obesity paradox.

There are some limitations to this meta-analysis. Firstly, all studies included were case-control studies with inherent selection, information and confounding biases. Secondly, the sample size was moderate for the included studies and a few of the eligible studies had small sample sizes. Thirdly, changes in testing methods and diagnostic criteria over time may have contributed to the different pooled results between publication years in the subgroup analysis.

In conclusion, the results of this meta-analysis suggest high levels of resistin levels are associated with an increased risk of SAP, indicating resistin may be a potential biomarker. Moreover, serum or plasma samples can be easily obtained for resistin detection, and the assay is uncomplicated and can be performed in many laboratories. Since it is often challenging for a single indicator to accurately predict the severity of AP, it may be possible in the future to predict SAP by testing for the levels of resistin in conjunction with other indicators or by incorporating resistin into a scoring system.

### Electronic supplementary material

Below is the link to the electronic supplementary material.

**Supplementary Material 1:** Forest plots of subgroup analysis by year of publication (A), age (B), sample size (C), and definition of SAP group and MAP group (D) in resistin

**Supplementary Material 2:** Forest plots of subgroup analysis by year of publication (A), age (B), sample size (C), and definition of SAP group and MAP group (D) in leptin

**Supplementary Material 3:** Forest plots of subgroup analysis by year of publication (A), age (B), sample size (C), and definition of SAP group and MAP group (D) in adiponectin

**Supplementary Material 4:** Forest plots of SMD with 95% CI of peripheral blood levels of resistin excluding the studies of Kibar YI et al., Singh AK et al. and Langmead C et al

**Supplementary Material 5:** Forest plots of SMD with 95% CI of peripheral blood levels of leptin excluding the studies of Türkoğlu A et al

**Supplementary Material 6:** Begg’s funnel plot of peripheral blood levels of resistin (A), leptin (B), and adiponectin (C) levels between SAP patients and MAP patients

**Supplementary Material 7:** Egger’s publication bias plot of peripheral blood levels of resistin (A), leptin (B), and adiponectin (C) levels between SAP patients and MAP patients

**Supplementary Material 8:** Search strategy in PubMed

**Supplementary Material 9:** Characteristics of 20 studies included in the meta-analysis (2)

**Supplementary Material 10:** Characteristics of 20 studies included in the meta-analysis (3)

**Supplementary Material 11:** Characteristics of 20 studies included in the meta-analysis (4)

**Supplementary Material 12:** The quality assessment of all included studies applying Quality Assessment of Diagnostic Accuracy Studies tool (QUADAS)

## Data Availability

All data generated or analyzed during this study are included in this published article and its supplementary information files.
