# Supplementary figures and images for: Predictive value of adipokines for the severity of acute pancreatitis: a meta-analysis
Source: BMC Gastroenterol. 2024 Jan 13;24:32. doi: 10.1186/s12876-024-03126-w (PMC10787974; doi:10.1186/s12876-024-03126-w)

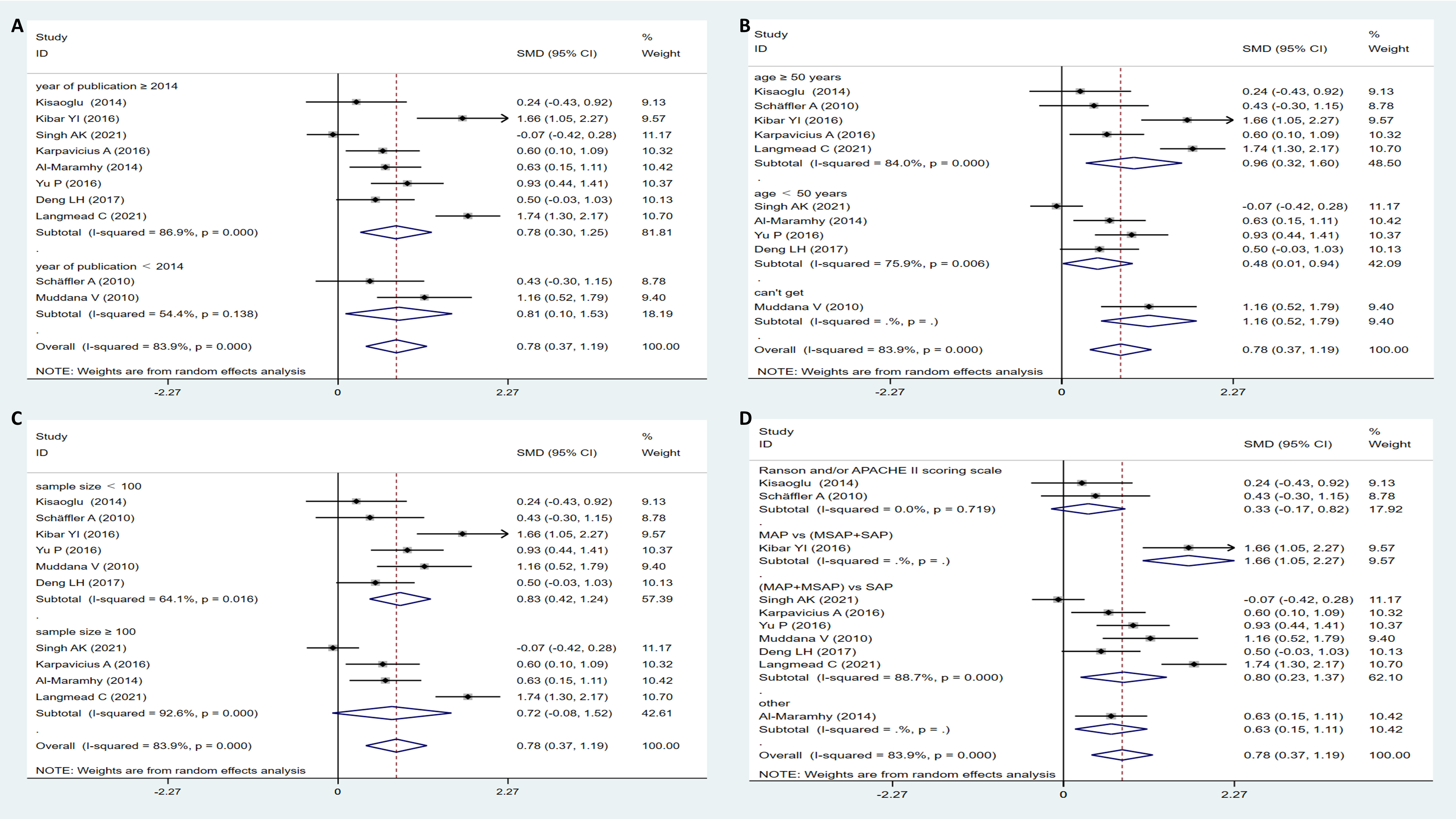

Supplement: Supplementary file 1 — Supplementary Material 1: Forest plots of subgroup analysis by year of publication (A), age (B), sample size (C), and definition of SAP group and MAP group (D) in resistin [file 12876_2024_3126_MOESM1_ESM.tif]

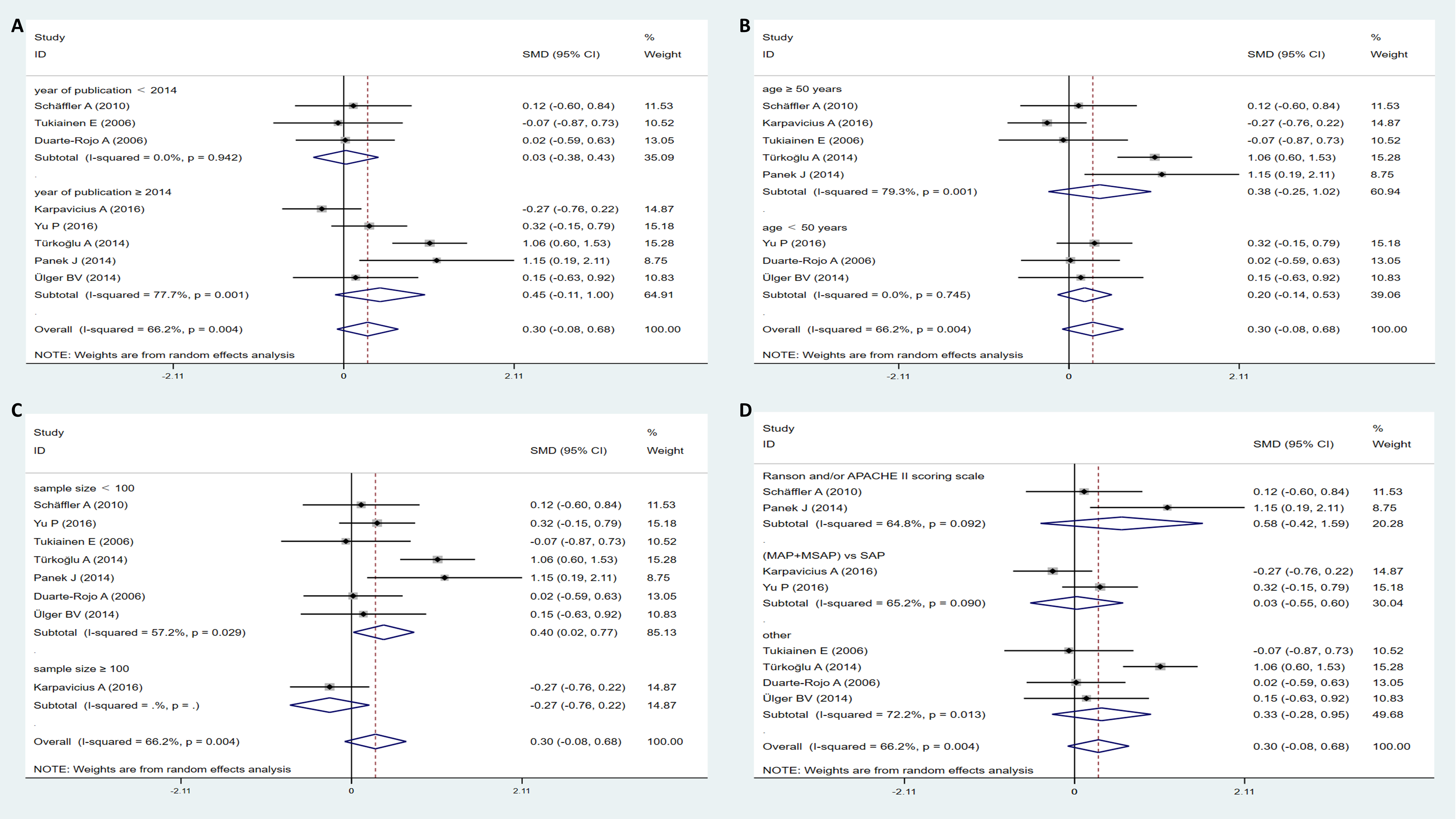

Supplement: Supplementary file 2 — Supplementary Material 2: Forest plots of subgroup analysis by year of publication (A), age (B), sample size (C), and definition of SAP group and MAP group (D) in leptin [file 12876_2024_3126_MOESM2_ESM.tif]

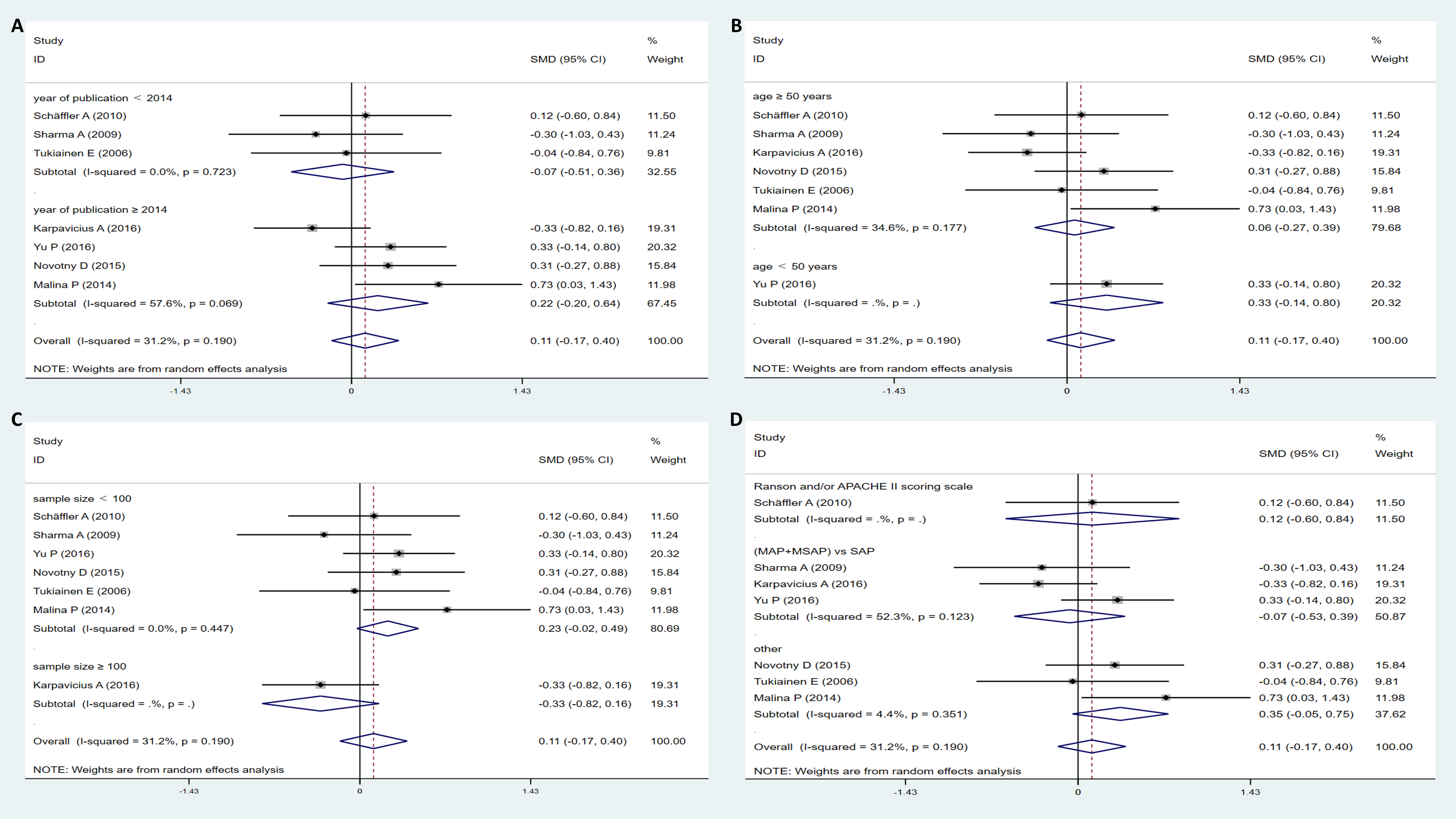

Supplement: Supplementary file 3 — Supplementary Material 3: Forest plots of subgroup analysis by year of publication (A), age (B), sample size (C), and definition of SAP group and MAP group (D) in adiponectin [file 12876_2024_3126_MOESM3_ESM.tif]

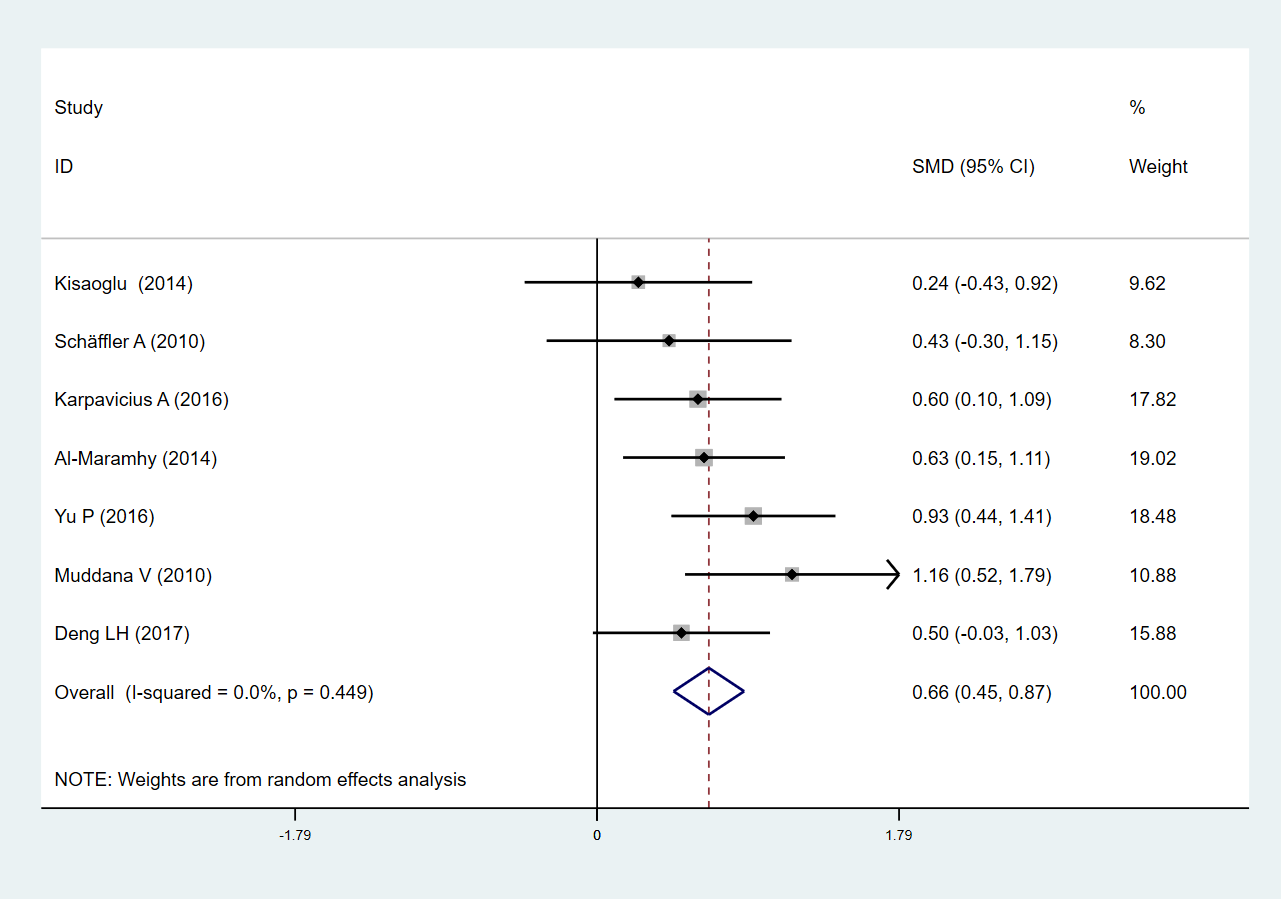

Supplement: Supplementary file 4 — Supplementary Material 4: Forest plots of SMD with 95% CI of peripheral blood levels of resistin excluding the studies of Kibar YI et al., Singh AK et al. and Langmead C et al [file 12876_2024_3126_MOESM4_ESM.tif]

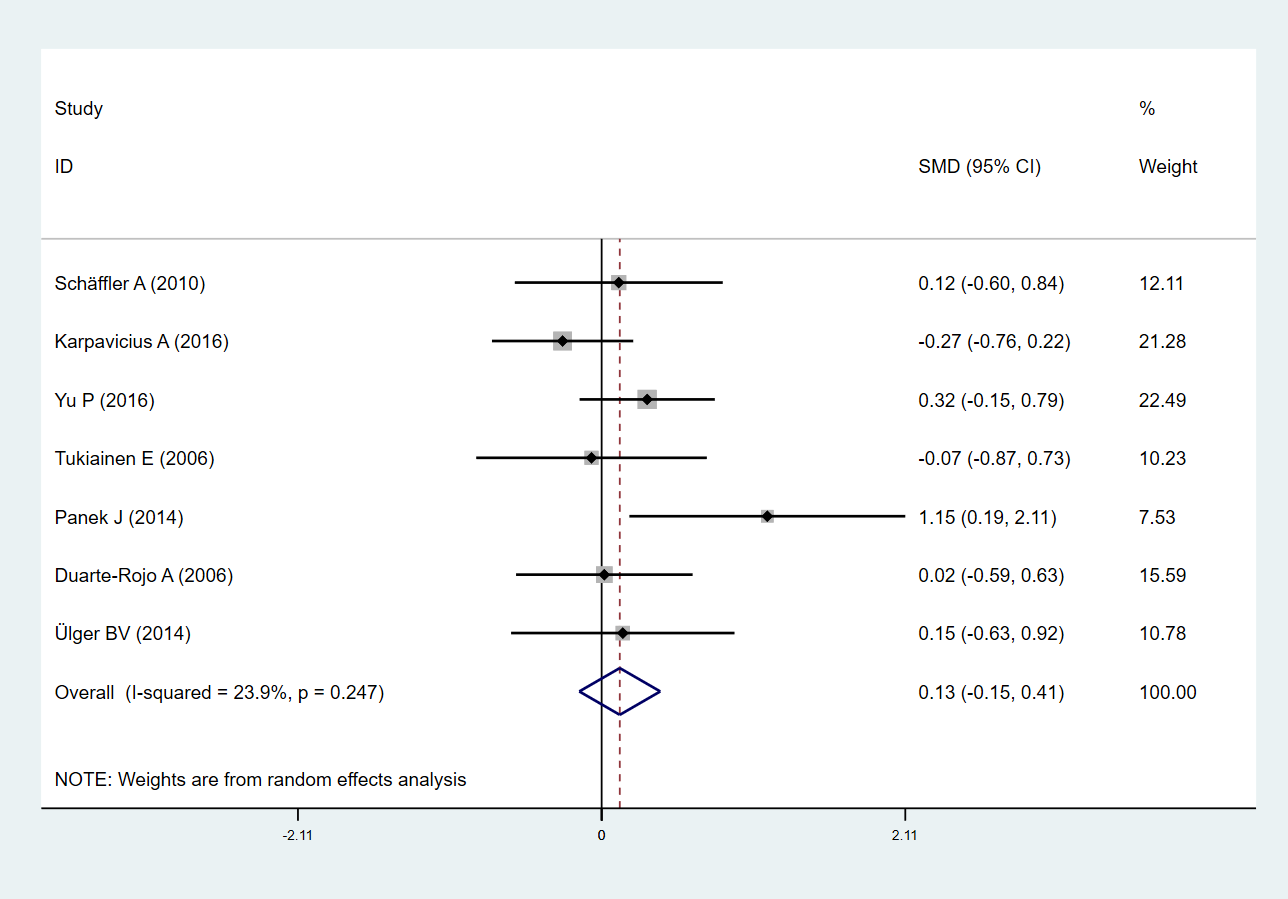

Supplement: Supplementary file 5 — Supplementary Material 5: Forest plots of SMD with 95% CI of peripheral blood levels of leptin excluding the studies of Türkoğlu A et al [file 12876_2024_3126_MOESM5_ESM.tif]

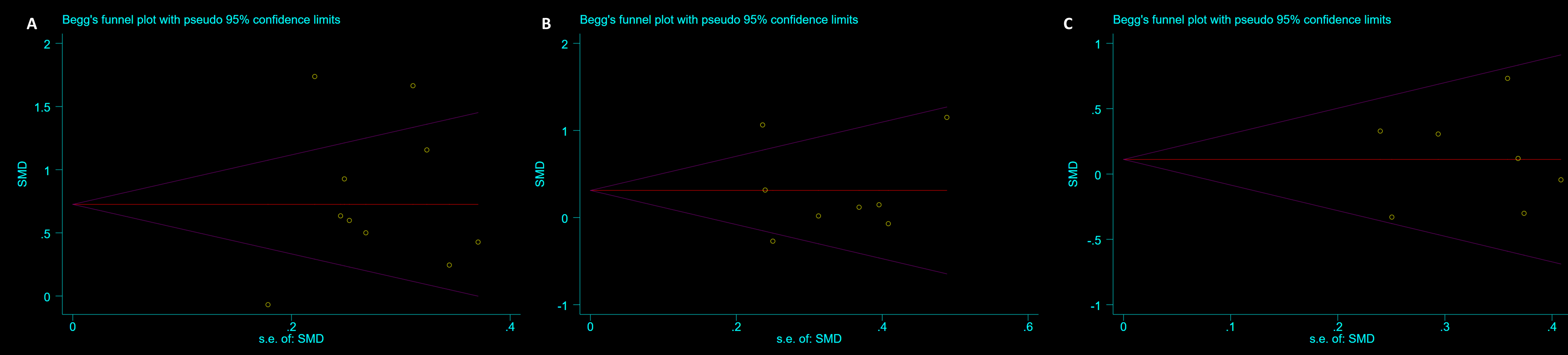

Supplement: Supplementary file 6 — Supplementary Material 6: Begg’s funnel plot of peripheral blood levels of resistin (A), leptin (B), and adiponectin (C) levels between SAP patients and MAP patients [file 12876_2024_3126_MOESM6_ESM.tif]

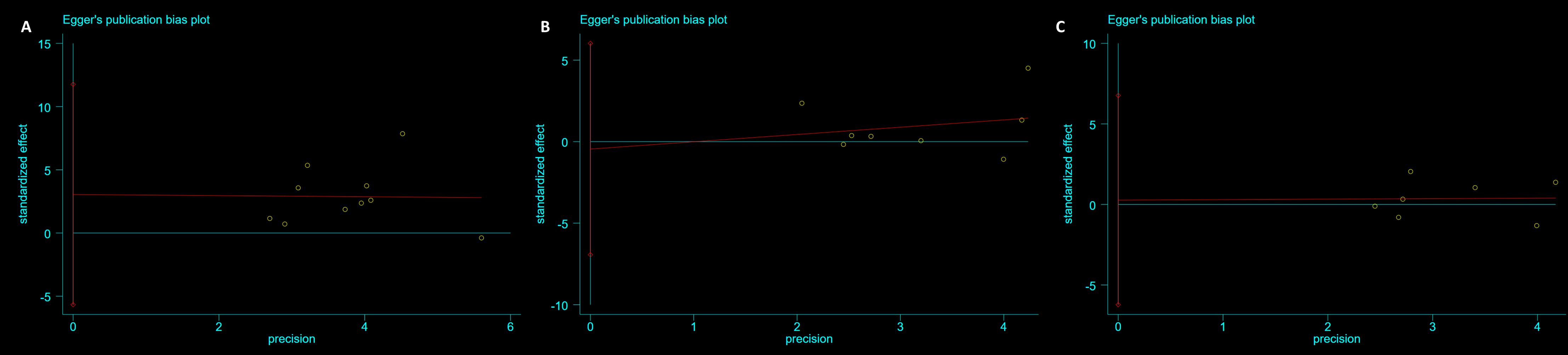

Supplement: Supplementary file 7 — Supplementary Material 7: Egger’s publication bias plot of peripheral blood levels of resistin (A), leptin (B), and adiponectin (C) levels between SAP patients and MAP patients [file 12876_2024_3126_MOESM7_ESM.tif]
