# Supplementary material for: Predictive value of adipokines for the severity of acute pancreatitis: a meta-analysis
Source: BMC Gastroenterol. 2024 Jan 13;24:32. doi: 10.1186/s12876-024-03126-w (PMC10787974; doi:10.1186/s12876-024-03126-w)
Supplement: Supplementary file 8 — Supplementary Material 8: Search strategy in PubMed [file 12876_2024_3126_MOESM8_ESM.docx]

**Table S1 Search strategy in PubMed**

| Search number | Query | Results |
| --- | --- | --- |
| 14 | (((((((("Resistin"[Mesh]) OR (Resistin[Title/Abstract])) OR (Adipocyte Cysteine-Rich Secreted Protein FIZZ3[Title/Abstract])) OR (Adipocyte Cysteine Rich Secreted Protein FIZZ3[Title/Abstract])) OR (((((((("Leptin"[Mesh]) OR (Leptin[Title/Abstract])) OR (Obese Protein[Title/Abstract])) OR (Obese Gene Product[Title/Abstract])) OR (Gene Product, Obese[Title/Abstract])) OR (Ob Gene Product Ob[Title/Abstract])) OR (Gene Product, Ob[Title/Abstract])) OR (Ob Protein[Title/Abstract]))) OR ((((((((("Adiponectin"[Mesh]) OR (Adiponectin[Title/Abstract])) OR (Adipocyte Complement-Related Protein 30-kDa[Title/Abstract])) OR (Adipocyte Complement Related Protein 30 kDa[Title/Abstract])) OR (Adipose Most Abundant Gene Transcript 1[Title/Abstract])) OR (apM-1 Protein[Title/Abstract])) OR (apM 1 Protein[Title/Abstract])) OR (ACRP30 Protein[Title/Abstract])) OR (Adipocyte, C1q[Title/Abstract] AND Collagen Domain Containing Protein[Title/Abstract]))) OR (((((((((("Nicotinamide Phosphoribosyltransferase"[Mesh]) OR (Nicotinamide Phosphoribosyltransferase[Title/Abstract])) OR (Phosphoribosyltransferase, Nicotinamide[Title/Abstract])) OR (NAMPT Protein[Title/Abstract])) OR (Pre-B-Cell Colony-Enhancing Factor[Title/Abstract])) OR (Colony-Enhancing Factor, Pre-B-Cell[Title/Abstract])) OR (Pre B Cell Colony Enhancing Factor[Title/Abstract])) OR (Visfatin[Title/Abstract])) OR (NAmPRTase[Title/Abstract])) OR (NMN Pyrophosphorylase NMN[Title/Abstract]))) OR ((((("Adipokines"[Mesh]) OR (Adipokines[Title/Abstract])) OR (Adipokine[Title/Abstract])) OR (Adipocytokine[Title/Abstract])) OR (Adipocytokines[Title/Abstract]))) AND ((((((((((((((((((((("Pancreatitis"[Mesh]) OR (Pancreatitis[Title/Abstract])) OR (Pancreatitis, Acute Edematous[Title/Abstract])) OR (Acute Edematous Pancreatitides[Title/Abstract])) OR (Edematous Pancreatitides, Acute[Title/Abstract])) OR (Edematous Pancreatitis, Acute[Title/Abstract])) OR (Pancreatitides, Acute Edematous[Title/Abstract])) OR (Acute Edematous Pancreatitis[Title/Abstract])) OR (Pancreatic Parenchymal Edema[Title/Abstract])) OR (Edema, Pancreatic Parenchymal[Title/Abstract])) OR (Pancreatic Parenchymal Edemas[Title/Abstract])) OR (Parenchymal Edema, Pancreatic[Title/Abstract])) OR (Pancreatic Parenchyma with Edema[Title/Abstract])) OR (Pancreatitis, Acute[Title/Abstract])) OR (Acute Pancreatitis[Title/Abstract])) OR (Acute Pancreatitides[Title/Abstract])) OR (Pancreatitides, Acute[Title/Abstract])) OR (Peripancreatic Fat Necrosis[Title/Abstract])) OR (Fat Necrosis, Peripancreatic[Title/Abstract])) OR (Necrosis, Peripancreatic Fat[Title/Abstract])) OR (Peripancreatic Fat Necroses[Title/Abstract])) | 287 |
| 13 | ((((((("Resistin"[Mesh]) OR (Resistin[Title/Abstract])) OR (Adipocyte Cysteine-Rich Secreted Protein FIZZ3[Title/Abstract])) OR (Adipocyte Cysteine Rich Secreted Protein FIZZ3[Title/Abstract])) OR (((((((("Leptin"[Mesh]) OR (Leptin[Title/Abstract])) OR (Obese Protein[Title/Abstract])) OR (Obese Gene Product[Title/Abstract])) OR (Gene Product, Obese[Title/Abstract])) OR (Ob Gene Product Ob[Title/Abstract])) OR (Gene Product, Ob[Title/Abstract])) OR (Ob Protein[Title/Abstract]))) OR ((((((((("Adiponectin"[Mesh]) OR (Adiponectin[Title/Abstract])) OR (Adipocyte Complement-Related Protein 30-kDa[Title/Abstract])) OR (Adipocyte Complement Related Protein 30 kDa[Title/Abstract])) OR (Adipose Most Abundant Gene Transcript 1[Title/Abstract])) OR (apM-1 Protein[Title/Abstract])) OR (apM 1 Protein[Title/Abstract])) OR (ACRP30 Protein[Title/Abstract])) OR (Adipocyte, C1q[Title/Abstract] AND Collagen Domain Containing Protein[Title/Abstract]))) OR (((((((((("Nicotinamide Phosphoribosyltransferase"[Mesh]) OR (Nicotinamide Phosphoribosyltransferase[Title/Abstract])) OR (Phosphoribosyltransferase, Nicotinamide[Title/Abstract])) OR (NAMPT Protein[Title/Abstract])) OR (Pre-B-Cell Colony-Enhancing Factor[Title/Abstract])) OR (Colony-Enhancing Factor, Pre-B-Cell[Title/Abstract])) OR (Pre B Cell Colony Enhancing Factor[Title/Abstract])) OR (Visfatin[Title/Abstract])) OR (NAmPRTase[Title/Abstract])) OR (NMN Pyrophosphorylase NMN[Title/Abstract]))) OR ((((("Adipokines"[Mesh]) OR (Adipokines[Title/Abstract])) OR (Adipokine[Title/Abstract])) OR (Adipocytokine[Title/Abstract])) OR (Adipocytokines[Title/Abstract])) | 69,742 |
| 12 | (((("Adipokines"[Mesh]) OR (Adipokines[Title/Abstract])) OR (Adipokine[Title/Abstract])) OR (Adipocytokine[Title/Abstract])) OR (Adipocytokines[Title/Abstract]) | 50,040 |
| 11 | ((((((((("Nicotinamide Phosphoribosyltransferase"[Mesh]) OR (Nicotinamide Phosphoribosyltransferase[Title/Abstract])) OR (Phosphoribosyltransferase, Nicotinamide[Title/Abstract])) OR (NAMPT Protein[Title/Abstract])) OR (Pre-B-Cell Colony-Enhancing Factor[Title/Abstract])) OR (Colony-Enhancing Factor, Pre-B-Cell[Title/Abstract])) OR (Pre B Cell Colony Enhancing Factor[Title/Abstract])) OR (Visfatin[Title/Abstract])) OR (NAmPRTase[Title/Abstract])) OR (NMN Pyrophosphorylase NMN[Title/Abstract]) | 3,188 |
| 10 | (((((((("Adiponectin"[Mesh]) OR (Adiponectin[Title/Abstract])) OR (Adipocyte Complement-Related Protein 30-kDa[Title/Abstract])) OR (Adipocyte Complement Related Protein 30 kDa[Title/Abstract])) OR (Adipose Most Abundant Gene Transcript 1[Title/Abstract])) OR (apM-1 Protein[Title/Abstract])) OR (apM 1 Protein[Title/Abstract])) OR (ACRP30 Protein[Title/Abstract])) OR (Adipocyte, C1q[Title/Abstract] AND Collagen Domain Containing Protein[Title/Abstract]) | 24,882 |
| 9 | ((((((("Leptin"[Mesh]) OR (Leptin[Title/Abstract])) OR (Obese Protein[Title/Abstract])) OR (Obese Gene Product[Title/Abstract])) OR (Gene Product, Obese[Title/Abstract])) OR (Ob Gene Product Ob[Title/Abstract])) OR (Gene Product, Ob[Title/Abstract])) OR (Ob Protein[Title/Abstract]) | 42,519 |
| 8 | ((("Resistin"[Mesh]) OR (Resistin[Title/Abstract])) OR (Adipocyte Cysteine-Rich Secreted Protein FIZZ3[Title/Abstract])) OR (Adipocyte Cysteine Rich Secreted Protein FIZZ3[Title/Abstract]) | 5,176 |
| 7 | (((((((((((((((((((("Pancreatitis"[Mesh]) OR (Pancreatitis[Title/Abstract])) OR (Pancreatitis, Acute Edematous[Title/Abstract])) OR (Acute Edematous Pancreatitides[Title/Abstract])) OR (Edematous Pancreatitides, Acute[Title/Abstract])) OR (Edematous Pancreatitis, Acute[Title/Abstract])) OR (Pancreatitides, Acute Edematous[Title/Abstract])) OR (Acute Edematous Pancreatitis[Title/Abstract])) OR (Pancreatic Parenchymal Edema[Title/Abstract])) OR (Edema, Pancreatic Parenchymal[Title/Abstract])) OR (Pancreatic Parenchymal Edemas[Title/Abstract])) OR (Parenchymal Edema, Pancreatic[Title/Abstract])) OR (Pancreatic Parenchyma with Edema[Title/Abstract])) OR (Pancreatitis, Acute[Title/Abstract])) OR (Acute Pancreatitis[Title/Abstract])) OR (Acute Pancreatitides[Title/Abstract])) OR (Pancreatitides, Acute[Title/Abstract])) OR (Peripancreatic Fat Necrosis[Title/Abstract])) OR (Fat Necrosis, Peripancreatic[Title/Abstract])) OR (Necrosis, Peripancreatic Fat[Title/Abstract])) OR (Peripancreatic Fat Necroses[Title/Abstract]) | 86,737 |
| 6 | "Adipokines"[Mesh] | 42,462 |
| 5 | "Nicotinamide Phosphoribosyltransferase"[Mesh] | 2,108 |
| 4 | "Leptin"[Mesh] | 26,106 |
| 3 | "Adiponectin"[Mesh] | 14,204 |
| 2 | "Resistin"[Mesh] | 2,657 |
| 1 | "Pancreatitis"[Mesh] | 56,584 |
