# Supplementary material for: Predictive value of adipokines for the severity of acute pancreatitis: a meta-analysis
Source: BMC Gastroenterol. 2024 Jan 13;24:32. doi: 10.1186/s12876-024-03126-w (PMC10787974; doi:10.1186/s12876-024-03126-w)
Supplement: Supplementary file 9 — Supplementary Material 9: Characteristics of 20 studies included in the meta-analysis (2) [file 12876_2024_3126_MOESM9_ESM.docx]

**Table S2 Characteristics of 20 studies included in the meta-analysis (2).**

| Author, year | Sex, n,  male(female) | | Age, year,  mean or median | | BMI kg/m2, mean or median | | Fund |
| --- | --- | --- | --- | --- | --- | --- | --- |
|  | SAP | MAP | SAP | MAP | SAP | MAP |  |
| Kisaoglu, 2014 | n/a | n/a | n/a | n/a | n/r | n/r | n/r |
| Schäffler A, 2010 | 23(18) | 9(1) | 57 | 30 | 60.8 | 30.4 | None |
| Kibar YI, 2016 | 9（13） | 12（25） | 54.8 | 63.7 | n/r | n/r | None |
| Singh AK, 2021 | n/a | n/a | n/a | n/a | 23.83 | 22.19 | None |
| Karpavicius A, 2016 | 12（8） | 38（44） | 55 | 56 | 31.1 | 28.0 | Supported by Vilnius University |
| Al-Maramhy, 2014 | n/a | n/a | n/a | n/a | n/a | n/a | Supported by Taibah University |
| Yu P,  2016 | n/a | n/a | n/a | n/a | n/a | n/a | National Natural Science Foundation of China |
| Muddana V, 2010 | n/r | n/r | n/r | n/r | n/r | n/r | n/r |
| Novotny D, 2015 | 10（4） | 37（33） | 56.1 | 58.7 | 30.40 | 26.93 | Supported by Internal Grant Agency of Palacky University Olomouc, Czech Republic |
| Sharma A, 2009 | n/a | n/a | n/a | n/a | n/a | n/a | Supported by the National Institute of Diabetes and Digestive and Kidney Diseases |
| Tukiainen E, 2006 | 8（4） | 8（4） | 55 | 54 | 27 | 26 | Grants from the Helsinki University Central Hospital Research Funds |
| Türkoğlu A, 2014 | 6(24) | 16(46) | 53.9 | 51.8 | 29.67 | 24.34 | Supported by Projects of Dicle University |
| Panek J, 2014 | 5（6） | 6（3） | 55 | 53 | 26.9 | 28.1 | Supported by a grant from State Committee for Scientific Research, Poland |
| Duarte-Rojo A, 2006 | 7（7） | 16（22） | n/a | n/a | 27 | 27 | n/r |
| Schäffler A, 2011 | 23(18) | 9(1) | 57 | 30 | 60.8 | 30.4 | None |
| Ülger BV,  2014 | n/r | n/r | 79.00 | 42.67 | n/r | n/r | Supported by the Coordinating Authority of Scientific Research Projects of Dicle University |
| Deng LH,  2017 | 17(3) | 37(13) | 43.5 | 43.2 | 25.85 | 25.32 | Supported by the National Natural Science Foundation of China |
| Langmead C,  2021 | n/a | n/a | n/a | n/a | n/a | n/a | Funded by the Department of Veterans Affairs Merit Review, the U.S. Department of Defense Congressionally Directed Medical Research Programs (CDMRP) Awards. |
| Malina P,  2014 | n/a | n/a | n/a | n/a | n/a | n/a | n/r |
| Guo F,  2021 | 16/14 | 19/16 | 48.72 | 47.38 | n/r | n/r | n/r |

n/a = not applicable; n/r = not reported
