# Supplementary material for: Predictive value of adipokines for the severity of acute pancreatitis: a meta-analysis
Source: BMC Gastroenterol. 2024 Jan 13;24:32. doi: 10.1186/s12876-024-03126-w (PMC10787974; doi:10.1186/s12876-024-03126-w)
Supplement: Supplementary file 10 — Supplementary Material 10: Characteristics of 20 studies included in the meta-analysis (3) [file 12876_2024_3126_MOESM10_ESM.docx]

**Table S3 Characteristics of 20 studies included in the meta-analysis (3).**

| Author, year | Diagnostic criteria for AP | Specific details of AP diagnostic criteria |
| --- | --- | --- |
| Kisaoglu, 2014 | Clinical, laboratory, and radiological findings | 1. Acute upper abdominal pain suggesting the clinical progression; 2. Hyperamylasemia of 5 items higher than the normal upper threshold; 3. Characteristic computer tomography and/or sonography findings. |
| Schäffler A, 2010 | Clinical, laboratory, and radiological findings | n/r |
| Kibar YI, 2016 | Clinic and laboratory findings | The presence of clinical findings, typical history, a more than three-fold increase in serum amylase and lipase values. |
| Singh AK, 2021 | Revised Atlanta criteria | The presence of at least 2 of the following 3 features: pain abdomen consistent with AP, raised serum lipase (or amylase) at least Accepted Manuscript3 times the upper limit of normal (ULN), and characteristic findings of AP on trans-abdominal USG, contrast enhanced CT abdomen or MRI abdomen |
| Karpavicius A, 2016 | Revised Atlanta criteria | Based on the presence of at least two of the three following features: abdominal pain characteristic of acute pancreatitis, serum amylase level ≥3 times up the upper limit of normal and characteristic findings of AP on abdominal computerized tomography scan. |
| Al-Maramhy, 2014 | Clinical, laboratory, and radiological findings | The diagnosis of AP was based on acute upper abdominal pain with an elevated serum amylase of at least three times the upper normal limit, which peaks approximately 24 h after the onset of attack and/or serum lipase. Gallstone pancreatitis was diagnosed based on radiologic findings of a gallstone or bile duct dilatation by abdominal ultrasonography and liver biochemical tests including serum alanine transaminase (≥ 65 U /L), serum bilirubin level (> 18.1 Umol/L), and alkaline phosphatase (> 137 U/L). |
| Yu, 2016 | Clinical, laboratory, and radiological findings | AP was diagnosed when two or more of the following were present: 1) abdominal pain characteristic of AP, 2) serum amylase and/or lipase 3 times the normal upper limit and 3) characteristic findings of AP on a CT scan. |
| Muddana V, 2010 | n/r | n/r |
| Novotny D, 2015 | Revised Atlanta classification | The study was performed on subjects with acute pancreatitis diagnosed according to the revised Atlanta classification from 2012. Diagnosis of acute pancreatitis was based on clinical, laboratory, and radiological findings during CT examination. |
| Sharma A, 2009 | Clinical, laboratory, and radiological findings | The diagnosis of AP was established if 2 of the following 3 criteria were present: (1) sudden onset of upper abdominal pain and/or abdominal localizing signs characteristic of AP; (2) serum amylase and/or lipase elevation at least 3 times the upper limit of normal; and (3) findings on abdominal imaging studies (eg, computed tomography) are consistent with AP. |
| Tukiainen E, 2006 | Clinical, laboratory, and radiological findings | The diagnosis of AP was based on typical clinical signs of abdominal pain, vomiting, or nausea; plasma amylase of more than 3 times the upper normal limit; and/or positive computed tomography scan. |
| Türkoğlu A, 2014 | Clinical, laboratory | The diagnosis of AP was made on admittance in the emergency department based on a history of acute abdominal pain with a 3-fold increase of serum amylase activity. |
| Panek J, 2014 | Clinical, laboratory, and radiological findings | The diagnosis of acute biliary pancreatitis (ABP) was based on clinical symptoms, elevated serum amylase activity (more than 3 times above the reference limit), abdominal ultrasonography (USG) and abdominal computed tomography (CT). |
| Duarte-Rojo A, 2006 | Clinical, laboratory, and radiological findings | Diagnosis of AP was based on typical clinical manifestations with at least a 3-fold increase of serum amylase and/or lipase. Whenever uncertainty about diagnosis existed, CT-scan was performed to confirm/rule out AP |
| Schäffler A, 2011 | Clinical, laboratory, and radiological findings during CT and / or ultrasound examination | n/r |
| Ülger BV,  2014 | Clinical, laboratory, and radiological findings | The patients were diagnosed with acute pancreatitis if they presented with two of the three following features: 1) abdominal pain characteristic of acute pancreatitis (acute epigastric pain that continues for more than 24 h), 2) serum amylase and/or lipase ≥3 times the upper limit of the normal, and 3) characteristic findings of acute pancreatitis on a computed tomography (CT) scan |
| Deng LH,  2017 | Revised Atlanta Classification | The diagnosis of AP requires 2 of the following 3 features: abdominal pain characteristic of AP; serum amylase and/or lipase activity at least 3 times greater than the reference limit; and findings characteristic of AP on abdominal computerized tomography scan or transabdominal ultrasonography |
| Langmead C,  2021 | American College of Gastroenterology guideline: management of acute pancreatitis | The diagnosis of AP is most oft en established by the presence of 2 of the 3 following criteria: (i) abdominal pain consistent with the disease, (ii) serum amylase and / or lipase greater than three times the upper limit of normal, and / or (iii) characteristic findings from abdominal imaging (strong recommendation, moderate quality of evidence). |
| Malina P,  2014 | History, clinical findings, and serum amylase levels | Based on history, clinical findings and at least a 5-fold increase of serum amylase levels against the upper range of the reference interval. |
| Guo F,  2021 | Chinese Guidelines for Diagnosis and Treatment of acute pancreatitis (Draft) | Clinically, it presents with acute, persistent abdominal pain, occasionally without abdominal pain, serum amylase activity is increased three times the upper limit of normal, and imaging suggests the pancreas with or without morphological changes to exclude other diseases. There may or may not be other organ dysfunction. In a minority of cases, serum amylase activity is normal or mildly elevated. |

n/r, not reported.
