# Supplementary material for: Predictive value of adipokines for the severity of acute pancreatitis: a meta-analysis
Source: BMC Gastroenterol. 2024 Jan 13;24:32. doi: 10.1186/s12876-024-03126-w (PMC10787974; doi:10.1186/s12876-024-03126-w)
Supplement: Supplementary file 11 — Supplementary Material 11: Characteristics of 20 studies included in the meta-analysis (4) [file 12876_2024_3126_MOESM11_ESM.docx]

**Table S4 Characteristics of 20 studies included in the meta-analysis (4).**

| Author, year | | Grouping criteria for MAP and SAP groups | Specific grouping details |
| --- | --- | --- | --- |
| Kisaoglu, 2014 | Ranson and APACHE II scoring scale | | Values ≥ 3 based on the Ranson scoring scale and values ≥ 8 in APACHE II scoring scale were considered to indicate severe AP. |
| Schäffler A, 2010 | Ranson and APACHE II scoring scale | | Values ≥ 3 based on the Ranson scoring scale and values ≥ 8 in APACHE II scoring scale were considered to indicate severe AP |
| Kibar YI,  2016 | Revision of the Atlanta classification. | | The first group included patients without complications (mild acute pancreatitis); the second group included patients with one or several organ failures (moderate or severe acute pancreatitis). |
| Singh AK, 2021 | Revisited Atlanta classification | | The first group included patients with MAP and MSAP; the second group included patients with SAP. |
| Karpavicius A, 2016 | Revisited Atlanta classification | | Based on organ failure, all the AP patients retrospectively were classified as mild, moderate or severe AP cases. The first group included patients with MAP and MSAP; the second group included patients with SAP. |
| Al-Maramhy, 2014 | Atlanta classification | | Severe AP was defined as the presence of at least one of the following criteria: pancreatic necrosis > 30%, pancreatic abscess, pseudocyst, systolic blood pressure < 90 mmHg, pO2 ≤60 mmHg, creatinine > 2 mg/dL after rehydration, gastrointestinal bleeding > 500 mL in 24 h, or death |
| Yu,  2016 | Modified Marshall scoring system | | Persistent organ failure was defined as organ failure that lasts for over 48 h Patients were divided into two groups: with and without persistent organ failure. |
| Muddana V, 2010 | Organ failure or not | | Severe AP (SAP) was defined by >1 organ dysfunction of cardiovascular, pulmonary and/or renal systems persisting for > 48 hours |
| Novotny D, 2015 | Disease severity score | | Mild/severe classification of AP (mild AP: 0 - 3 points; severe AP: 4 - 10 points) |
| Sharma A, 2009 | Organ failure or not | | Severe AP was defined as the presence of cardiovascular, pulmonary, and/or renal system dysfunction during the initial hospital admission for at least 48 hours. |
| Tukiainen E, 2006 | The criteria of Atlanta | | Severe acute pancreatitis is associated with organ failure and/or local complications, such as necrosis, abscess, or pseudocyst |
| Türkoğlu A, 2014 | The Atlanta Criteria | | Severe acute pancreatitis is associated with organ failure and/or local complications, such as necrosis, abscess, or pseudocyst |
| Panek J,  2014 | The Atlanta criteria | | Values ≥ 3 based on the Ranson scoring scale or values ≥ 8 in APACHE II scoring scale were considered to indicate severe AP |
| Duarte-Rojo A, 2006 | The Atlanta criteria | | Severe AP was considered when patients developed one or more local (i.e. necrosis, infected necrosis, abscess, pseudocyst) or systemic (i.e. renal or respiratory failure, cardiovascular collapse, coagulopathy, gastrointestinal bleeding, sepsis or multiple organ failure) complications according to the Atlanta classification of AP |
| Schäffler A, 2011 | Ranson score and APACHE-II  score | | Values ≥ 3 based on the Ranson scoring scale and values ≥ 8 in APACHE II scoring scale were considered to indicate severe AP |
| Ülger BV,  2014 | The presence of local complications or organ failure | | The patients were divided into mild or severe AP groups according to the presence of local complications or organ failure, respectively. Organ failure was defined as hypotension (systolic blood pressure ≤90 mmHg), respiratory system failure (arterial pO2 <60 mmHg), renal failure (blood creatinine >2 mg/dL), or gastrointestinal hemorrhage (>500 mL in 24 h). Local complications were pancreatic necrosis, abscess, or pseudocysts. |
| Deng LH,  2017 | Revisited Atlanta classification | | Mild AP was defined as the absence of both organ failure (OF) and local or systemic complications. Moderately severe AP was defined as the presence of transient OF or local or systemic complications in the absence of persistent OF. SAP was characterized by persistent OF. Using the Modified Marshall Scores (MMS), OF was defined as a score of 2 or more for 1 of 3 organ systems. Persistent OF was defined as OF for more than 48 hours. |
| Langmead C,  2021 | Revisited Atlanta classification | | The sample was divided into two groups based on the presence or absence of organ failure, which was defined using the Modified Marshall Scores |
| Malina P，  2014 | Computed tomography severity index (CTSI) | | Patients were divided into two groups - mild AP (CTSI score 0-5) and severe AP (CTSI score 6-10). |
| Guo F,  2021 | APACHE II score | | Patients were divided into mild acute pancreatitis (MAP) group and severe acute pancreatitis (SAP) group according to the acute physiology and chronic health II (APACHE II) score (< 8, ≥8 scores) |
