# Supplementary material for: Predictive value of adipokines for the severity of acute pancreatitis: a meta-analysis
Source: BMC Gastroenterol. 2024 Jan 13;24:32. doi: 10.1186/s12876-024-03126-w (PMC10787974; doi:10.1186/s12876-024-03126-w)
Supplement: Supplementary file 12 — Supplementary Material 12: The quality assessment of all included studies applying Quality Assessment of Diagnostic Accuracy Studies tool (QUADAS) [file 12876_2024_3126_MOESM12_ESM.docx]

**Table S5 The quality assessment of all included studies applying Quality Assessment of Diagnostic Accuracy Studies tool (QUADAS)**

| Author, year | ① | ② | ③ | ④ | ⑤ | ⑥ | ⑦ | ⑧ | ⑨ | ⑩ | ⑪ | ⑫ | ⑬ | ⑭ |
| --- | --- | --- | --- | --- | --- | --- | --- | --- | --- | --- | --- | --- | --- | --- |
| Kisaoglu, 2014 | √ | √ | √ | √ | √ | √ | √ | √ | √ | √ | √ | √ | ? | ? |
| Schäffler A, 2010 | √ | √ | √ | √ | √ | √ | √ | √ | √ | √ | √ | √ | ? | ? |
| Kibar YI, 2016 | √ | √ | √ | √ | √ | √ | √ | √ | √ | √ | √ | √ | ? | ? |
| Singh AK, 2021 | √ | √ | √ | √ | √ | √ | √ | √ | √ | √ | √ | √ | ? | √ |
| Karpavicius A, 2016 | √ | √ | √ | √ | √ | √ | √ | √ | √ | √ | √ | √ | ? | ? |
| Al-Maramhy, 2014 | - | √ | √ | √ | √ | √ | √ | √ | √ | √ | √ | - | ? | ? |
| Yu, 2016 | √ | √ | √ | √ | √ | √ | √ | √ | √ | √ | √ | √ | ? | ? |
| Muddana V, 2010 | ? | ? | √ | √ | √ | √ | √ | √ | √ | √ | √ | ? | ? | ? |
| Novotny D, 2015 | √ | √ | √ | √ | √ | √ | √ | √ | √ | √ | √ | √ | ? | ? |
| Sharma A, 2009 | √ | √ | √ | √ | √ | √ | √ | √ | √ | √ | √ | √ | ? | ? |
| Tukiainen E, 2006 | √ | √ | √ | √ | √ | √ | √ | √ | √ | √ | √ | √ | ? | ? |
| Türkoğlu A, 2014 | √ | √ | √ | √ | √ | √ | √ | √ | √ | √ | √ | √ | ? | √ |
| Panek J, 2014 | √ | √ | √ | √ | √ | √ | √ | √ | √ | √ | √ | √ | ? | ? |
| Duarte-Rojo A, 2006 | √ | √ | √ | √ | √ | √ | √ | √ | √ | √ | √ | √ | ? | √ |
| Schäffler A, 2011 | √ | √ | √ | √ | √ | √ | √ | √ | √ | √ | √ | √ | ? | √ |
| Ülger BV, 2014 | - | √ | √ | √ | √ | √ | √ | √ | √ | √ | √ | - | ? | ? |
| Deng LH,  2017 | √ | √ | √ | √ | √ | √ | √ | √ | √ | √ | √ | √ | ? | ? |
| Langmead C,  2021 | √ | √ | √ | √ | √ | √ | √ | √ | √ | √ | √ | √ | ? | √ |
| Malina P,  2014 | √ | √ | √ | √ | √ | √ | √ | √ | √ | √ | √ | √ | ? | ? |
| Guo F,  2021 | ? | √ | √ | √ | √ | √ | √ | √ | √ | √ | √ | ? | ? | ? |

√, yes; -, no; ?, unclear; ①, Does the disease spectrum include a variety of cases; ②, Whether the criteria for the selection of research subjects are clear; ③, Can the gold standard differentiate the severity of AP; ④, Whether the interval between the gold standard and the test to be evaluated is sufficiently short to avoid a change in the disease condition (disease progression bias); ⑤, Whether all samples or randomly selected samples were subjected to gold standard tests (partial reference bias); ⑥, Whether all patients received the same gold-standard test regardless of the outcome of the trial to be evaluated (multiple reference bias); ⑦, Whether the gold standard test is independent of the test to be evaluated (mixed bias); ⑧, Are the operations of the test to be evaluated sufficiently clearly described and repeatable (conduct of the test to be evaluated); ⑨, Is the operation of the gold standard test sufficiently clearly described and repeatable (implementation of the Gold Standard); ⑩, Whether the outcome interpretation of the trial to be evaluated was performed without knowledge of the results of the gold standard trial (trial interpretation bias); ⑪, Whether the interpretation of gold standard test results was performed without knowledge of the results of the trial to be evaluated (gold standard interpretation bias); ⑫, Whether the clinical information available when interpreting the results is one to the clinical information available in actual use (clinical interpretation bias); ⑬, Are difficult to interpret/intermediate test results reported; ⑭, Explanation of cases withdrawn from the study or not.
